# Risk factors of tubal infertility in a tertiary hospital in a low-resource setting: a case-control study

**DOI:** 10.1186/s40738-020-00073-4

**Published:** 2020-03-06

**Authors:** Thomas Obinchemti Egbe, Theophile Nana-Njamen, Felix Elong, Robert Tchounzou, Andre Gaetan Simo, Gaelle Padjip Nzeuga, Cedric Njamen Nana, Emmanuella Manka’a, Charlotte Tchente Nguefack, Gregory Edie Halle-Ekane

**Affiliations:** 1 Department of Obstetrics and Gynecology, Douala Referral Hospital, Douala, Cameroon; 2 Faculty of Health Sciences, University of Buea, Buea, Cameroon; 3 Faculty of Health Sciences, Université des Montagnes, Bangangte, Cameroon; 4 Department of Radiology, Douala Referral Hospital, Douala, Cameroon; 5 Faculty of Medicine and Pharmaceutical Sciences, University of Douala, Douala, Cameroon

**Keywords:** Tubal infertility, Associated risk factors, Pelvic inflammatory disease, Sexually transmitted infections

## Abstract

**Background:**

Infertility is the inability to sustain a pregnancy in a woman with regular (2–3 times per week) unprotected sexual intercourse for a period of 1 year. This is a major public health problem that remains under-recognised in Cameroon and most countries in sub-Saharan Africa. This study aimed at identifying the risk factors associated with tubal infertility in a tertiary hospital in Douala, Cameroon.

**Methods:**

We conducted a case-control study at the Obstetrics, Gynaecology and Radiology Departments of the Douala Referral Hospital from October 1, 2016, to July 30, 2017. We recruited 77 women with tubal infertility diagnosed using hysterosalpingography and 154 unmatched pregnant women served as controls. Data on socio-demographic, reproductive and sexual health, and radiologic assessments were collected using a pretested questionnaire. The data were analysed using the Statistical Package for the Social Sciences (SPSS) software version 24.0. Logistic regression models were fitted to identify demographic, reproductive health factors, surgical, medical and toxicological factors associated with tubal infertility. The adjusted odds ratios (AOR) and their 95% confidence interval were interpreted. Statistical significance set at *p* < 0.05.

**Results:**

Sixty-one per cent of respondents had secondary infertility. Following multivariate logistic regression analysis, respondents who were housewives (AOR 10.7; 95% CI: 1.68–8.41, *p* = 0.012), self-employed (AOR 17.1; 95% CI: 2.52–115.8, *p* = 0.004), with a history of *Chlamydia trachomatis* infection (AOR 17.1; 95% CI: 3.4–85.5, *p* = 0.001), with *Mycoplasma* infection (AOR 5.1; 95% CI: 1.19–22.02, *p* = 0.03), with ovarian cyst (AOR 20.5; 95% CI: 2.5–168.7, *p* = 0.005), with uterine fibroid (AOR 62.4; 95% CI: 4.8–803.2, *p* = 0.002), have undergone pelvic surgery (AOR 2.3; 95% CI: 1.0–5.5, *p* = 0.05), have undergone other surgeries (AOR 49.8; 95% CI: 6.2–400, *p* = 0.000), diabetic patients (AOR 10.5; 95% CI 1.0–113.4, *p* = 0.05) and those with chronic pelvic pain (AOR 7.3; 95% CI: 3.2–17.1, *p* = 0.000) were significantly associated with tubal infertility while the young aged from 15 to 25 (AOR 0.07; 95% CI: 0.01–0.67, 0.021), those in monogamous marriages (AOR 0.05; 95% CI: 0.003–1.02, *p* = 0.05), as well as those with a history of barrier contraceptive methods (condom) (AOR 0.17; 95% CI: 0.03–1.1, *p* = 0.06) were less likely to have tubal infertility.

**Conclusion:**

The following factors were independently associated with tubal infertility: being a housewife, self-employed, history of *Chlamydia trachomatis*, *Mycoplasma* infection, and uterine fibroid. Furthermore, a history of pelvic surgery and other surgeries, diabetes mellitus, and chronic pelvic pain were also associated with tubal infertility. Young age, persons in monogamous marriages and users of barrier methods of contraception (condom) were less likely to have tubal infertility. Identification of these factors will be a target of intervention to avoid tubal infertility.

## Background

Infertility is the inability to sustain a pregnancy in a woman with regular (2–3 times per week) unprotected sexual intercourse for a period of 1 year [1]. Though it is a major public health problem, infertility in sub-Saharan Africa remains largely under-recognised [2, 3]. An infertility belt has actually been described in Africa that cuts across West and Central Africa, including Cameroon [4]. Though the prevalence of infertility has been widely reported in medical literature, it is difficult to synthesize infertility prevalence data because of the incomparable definitions used [5]. However, in Africa, and Cameroon, in particular, this prevalence has been underestimated because infertile patients do not readily seek medical attention for various reasons including lack of awareness or knowledge, lack of resources as well as cultural and religious reasons [6–8]. It has been reported, previously, in Yaoundé, Cameroon that the female factor accounts for 30% of infertility; with infectious causes mainly Chlamydia, accounting for 48.9% [9]. There are few studies in Cameroon that report the risk factors associated with infertility. This study aimed at identifying the risk factors associated with tubal infertility at the Douala Referral Hospital, Cameroon.

## Patients and methods

### Study design and site

We conducted a case-control study from October 1, 2016 to July 30, 2017 at the Obstetrics and Gynaecology and Radiology Departments of the Douala Referral Hospital (DRH)). The DRH is a tertiary health facility that provides scientific treatment, research and teaching, and serves as a referral hospital for Douala and the Central African sub-region. The Department of Obstetrics and Gynaecology has eight obstetricians/gynaecologists while the Department of Radiology has three radiologists. Patients who were consulted by the gynaecologist were later referred to the radiologists for hysterosalpingography (HSG) after screening and treating for vaginal infections. We enrolled consenting women suffering from infertility and whose HSG results showed bilateral tubal occlusion. We excluded women with other causes of infertility like diminished ovarian reserve, male factor, uterine factor, or ovulatory factor. The sperm count of the partner was normal. The control group consisted of consenting women who became pregnant naturally and came for antenatal care visits during the study period.

### Study procedure

- After obtaining ethical approval from the research board of the DRH, respondents signed a written informed consent form to take part in the study. Women with a diagnosis of infertility and pregnant controls were administered pretested questionnaires consisting of:
- Sociodemographic information: age, marital status, level of education and occupation
- Reproductive health characteristics: age at first intercourse, contraception use and type, parity, number of lifetime sex partners, type of dysmenorrhea, histories of ectopic pregnancies, uterine fibroid, ovarian cysts, spontaneous and induced abortions, use of traditional vaginal herbs, type of infertility, duration of infertility, infection screening (*Chlamydia trachomatis*, *Mycoplasma*), history of PID and lesions found on hysterosalpingography (unilateral or bilateral tubal obstruction). For this study, we considered only bilateral tubal occlusion for tubal infertility
- Past medical, surgical and toxicological history: history of pelvic and other surgeries, diabetes mellitus, tobacco smoking

The HSG was performed on out-patient basis during the proliferative phase that is during the 7th–12th day of menstrual cycle (the 1st day being the menstrual bleeding and women with a regular cycle) using the standard technique [10–12]. All the procedures were performed at the DRH and reviewed for endometrial and tubal pathologies by three experienced radiologists to avoid inter-observer bias.

**Fig. 1:**
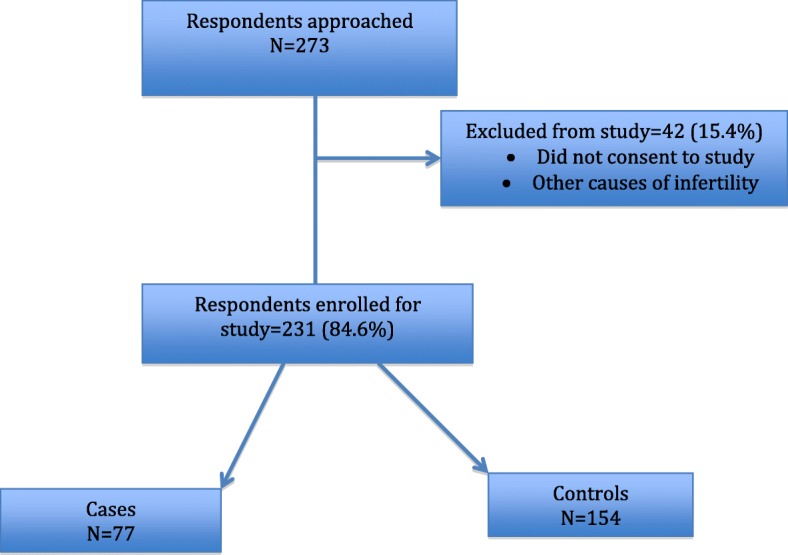
Flow diagram of study population

There were, 77 respondents with tubal factor infertility (cases) and 154 controls (women who came for antenatal care visits) (Fig. 1). Reporting was according to the Strokes guidelines.

### Data management and analysis

Data were coded and double entered into Microsoft excel 2013 by two separate researchers to avoid errors. The data were analysed using the Statistical Package for the Social Sciences (SPSS) software version 24.0. Logistic regression models were fitted to identify demographic, reproductive health factors, surgical, medical and toxicological factors associated with tubal infertility. The adjusted odds ratios (AOR) and their 95% confidence interval were interpreted. The threshold for type 1 error was set at 5%.

## Results

Figure 1 shows that we approached 273 respondents at the Obstetrics and Gynaecology Department of the DRH, among which 231 (84.6%) were enrolled for study and 42 (15.4%) were excluded from study because they did not consent to study or had other causes of infertility. Among this group, 77 had tubal factor infertility (cases) and 154 women with spontaneous pregnancies who came for antenatal care visits served as controls. Furthermore, 61% (47/77) of cases had secondary infertility. Respondent’s age ranged from 15 to 43 years with a mean age of 30.6 (SD 5.97) years.

Table 1 shows that there is no significant difference between the two groups with regard to those under the age of 35, level of education, region of origin, and most professions aside from self-employed (*p* < 0.001).

**Table 1:** Socio-demographic characteristics of study population

Variables	Frequency cases = 77		Frequency controls = 154		Odd ratios	95% CI	*P*-value
	*N*	%	*N*	%			
Age (years)							
15–25	1.0	1.3	37	24	0.07	0.09–0.51	0.09
25–35	55	71.4	96	62.3	0.67	0.38–1.19	0.171
35–45	21	27.3	21	13.6	3.64	1.92–6.88	< 0.001
Marital status							
Single	24	31.2	62	40.3	0.67	0.38–1.20	0.18
Cohabitation	14	18.2	1	0.6	34	4.38–264.07	0.001
Monogamy	37	48.1	91	59.1	0.66	0.38–1.14	0.136
Polygamy	2	2.6	1	0.6	4.08	0.36–45.71	0.254
Level of education							
Primary	28	36.4	46	29.9	0.86	0.44–1.67	0.66
Secondary	41	53.2	92	59.7	0.75	0.43–1.30	0.31
Tertiary	8	10.4	16	10.4	2.14	0.07–12.7	0.97
Profession							
Civil servant	29	37.7	54	35.1	1.12	0.63–1.97	0.698
Private sector	14	18.2	22	14.3	1.33	0.64–2.78	0.443
Student/pupil	15	19.5	13	8.4	2.62	1.18–5.84	0.018
Self-employed	3	3.9	40	1.9	0.12	0.034–0.39	< 0.001
Housewife	16	20.8	25	16.2	1.42	0.70–2.87	0.327
Region of origin							
Adamawa	2	2.6	4	2.6	1.34	0.22–8.21	0.75
Center	10	12.9	17	11.0	1.20	0.52–2.77	0.66
East	15	19.5	21	13.6	1.62	0.78–3.38	1.20
West	29	37.6	64	41.5	0.83	0.47–1.45	0.51
Northwest	1	1.3	3	1.9	0.66	0.07–6.47	0.72
Far North	1	1.3	4	2.6	0.49	0.05–4.49	0.53
North	2	2.6	6	3.9	0.66	0.13–3.34	0.61
South	7	9.1	13	8.4	1.09	0.41–2.84	0.87
Southwest	4	5.2	10	6.5	0.79	0.24–2.78	0.70
Littoral	6	7.8	12	7.8	1.00	0.36–2.78	1.00

*CI* Confidence interval, *N* Number, % Percentage

### Risk factors of tubal infertility (multivariate analysis)

Table 2 shows the socio-demographic factors associated with tubal infertility.

**Table 2:** Demographic factors associated with tubal infertility (multivariate analysis)

Variable	Levels	Cases	Control			
		*n* (%)	*n* (%)	AOR	95% CI	Sig.
Age	15–25	1 (0.4)	37 (16.1)	0.072	0.01–0.67	**0.021**
	26–35	56 (24.3)	94 (40.9)	0.592	0.27–1.31	0.195
	35–45	22 (9.6)	20 (8.7)	1		
Marital status	Single	25 (10.9)	61 (26.5)	0.067	0.003–1.38	0.080
	Cohabiting	14 (6.1)	1 (0.4)	1.995	0.06–66.79	0.700
	Monogamy	38 (6.1)	88 (38.3)	0.051	0.003–1.02	0.052
	Polygamy	2 (0.9)	1 (0.4)	1		
Level of education	Higher	28 (12.4)	31 (13.8)	1.734	0.82–0.67	0.151
	Primary	15 (6.7)	33 (14.7)	0.688	0.28–1.70	0.419
	Secondary	36 (16.0)	82 (36.4)	1		
Profession	Housewife	17 (7.4)	23 (10.0)	10.722	1.68–8.41	**0.012**
	Private sector	14 (6.1)	21 (9.2)	6.191	0.93–41.10	0.059
	Civil servant	30 (13.1)	53 (23.1)	5.451	0.92–32.35	0.062
	Self-employed	15 (6.6)	13 (5.7)	17.077	2.52–115.78	**0.004**
	Students	3 (1.3)	40 (17.5)	1		

The procedure models cases as the response, treating control as the reference category

*AOR* Adjusted odd ratio, *CI* Confidence interval

Respondents who were housewives (AOR 10.7; 95% CI: 1.68–8.41, *p* = 0.012) and those who were self-employed (AOR 17.1; 95% CI: 2.52–115.8, *p* = 0.004) were significantly associated with tubal infertility while young age 15–25 years (AOR 0.07; 95% CI: 0.01–0.67, 0.021) and those who married monogamously (AOR 0.05; 95% CI: 0.003–1.02, *p* = 0.05) were less likely to have tubal infertility.

Table 3 shows the reproductive health characteristics that were independently associated with tubal infertility.

**Table 3:** Reproductive health factors associated with tubal infertility (multivariate analysis)

Variable	Levels	Cases	Control	AOR	Lower	Upper	Sign.
		n (%)	n (%)				
Age at first intercourse	15–20	74 (32.2)	117 (50.9)	0.56	0.0	4.19	0.572
	21–35	5 (2.2)	34 (14.8)	1			
Type of Dysmenorrhoea	Secondary	74 (32.2)	129 (56.1)	1.40	0.09	20.65	0.808
	Primary	5 (2.2)	22 (9.)	1			
Given birth before	Yes	27 (12.1)	78 (34.8)	0.47	0.12	1.85	0.281
	No	46 (20.5)	73 (32.6)	1			
Use o condom	Yes	28 (12.2)	104 (45.4)	0.17	0.03	1.08	0.061
	No	50 (21.8)	47 (20.5)	1			
Number of sexual partners	> one	21 (9.1)	16 (7.0)	3.42	0.62	18.81	0.158
	One partner	58 (25.2)	135 (58.7)	1			
Ovarian Cyst	Yes	13 (5.7)	6 (2.6)	20.45	2.48	168.67	**0.005**
	No	66 (28.7)	145 (3.0)	1			
Induced abortion	Yes	28 (12.2)	39 (17.0)	2.75	0.64	11.81	0.173
	No	51 (22.2)	112 (48.7)	1			
Chlamydia	Yes	38 (16.5)	12 (5.2)	17.05	3.40	85.53	**0.001**
	No	41 (17.8)	139 (60.4)	1			
Mycoplasma	Yes	43 (18.7)	27 (11.7)	5.13	1.19	22.02	**0.028**
	No	36 (15.7)	124 (53.9)	1			
Deep dyspareunia	Yes	29 (12.6)	7 (3.0)	3.73	0.38	36.40	0.258
	No	50 (21.7)	144 (62.6)	1			
Multiple uterine fibroids	Yes	20 (8.7)	3 (1.3)	62.35	4.84	803.18	**0.002**
	No	59 (25.7)	148 (64.3)	1			

The procedure models cases as the response, treating control as the reference category

Respondents with a history of *Chlamydia trachomatis* infection (AOR 17.1; 95% CI: 3.4–85.5, *p* = 0.001), *Mycoplasma* infection (AOR 5.1; 95% CI: 1.19–22.02, *p* = 0.03), ovarian cyst (AOR 20.5; 95% CI: 2.5–168.7, *p* = 0.005) and those with uterine fibroids (AOR 62.4; 95% CI: 4.8–803.2, *p* = 0.002) were significantly associated with tubal infertility. However, respondents with a history of use of barrier contraceptive methods (condom) (AOR 0.17; 95% CI: 0.03–1.1, *p* = 0.06) were less likely to have tubal infertility during the study period.

Table 4 shows the medical and surgical factors independently associated with tubal infertility.

**Table 4:** Surgical and medical factors associated with tubal infertility (multivariate analysis)

				AOR	95% CI	Sig.
Other surgery	Yes	18 (9.0)	1 (0.5)	49.826	6.20–400.14	0.000
	No	52 (2.0)	129 (64.5)	1		.
Pelvic surgery	Yes	19 (8.3)	19 (8.3)	2.318	0.98–5.47	0.055
	No	60 (26.1)	132 (57.4)	1		.
Diabetes	Yes	3 (1.3)	1 (0.4)	10.494	0.97–113.38	0.053
	No	76 (33.0)	150 (65.2)	1		.
Chronic pelvic pains	Yes	35 (15.2)	11 (4.8)	7.331	3.15–17.05	0.000
	No	44 (19.1)	140 (60.9)	1	.	.

The procedure models cases as the response, treating control as the reference category

Participants who had undergone other surgeries (AOR 49.8; 95% CI: 6.2–400, *p* = 0.000), pelvic surgery (AOR 2.3; 95% CI: 1.0–5.5, *p* = 0.05), diabetic patients (AOR 10.5; 95% CI 1.0–113.4, *p* = 0.05) and those with chronic pelvic pain (AOR 7.3; 95% CI: 3.2–17.1, *p* = 0.000) were significantly associated with tubal infertility.

## Discussion

We conducted a case-control study that aimed at identifying the risk factors associated with tubal factor infertility at the Douala Referral Hospital, Cameroon.

The risk factors of tubal infertility were: being a housewife, self-employed, history of *Chlamydia trachomatis*, *Mycoplasma* infection, and uterine fibroids. Furthermore, the history of pelvic surgery and other surgeries, diabetes mellitus, and chronic pelvic pain were also associated with tubal infertility. Young age, those in monogamous marriages and users of barrier methods of contraception (condom) were less likely to have tubal infertility.

### Sociodemographic variables associated with tubal infertility

Sixty-one per cent of patients in the study had secondary infertility with 35.1% (27/77) being parous. Besides, other studies have reported that parity, however, may be an inappropriate substitute for infertility because it cannot account for pregnancy intention [13].

In this study, housewives and the self-employed respondents were prone to have tubal infertility. The reason for this may be associated with poor sexual habits of their partners. There is still free sex life among men and especially those engaged in liberal businesses in Cameroon. This is compounded with the low use of barrier family planning methods especially the condom. Some men believe that condom use reduces sexual pleasure and men who have this belief are less likely to use the condom [14]. The non-use of condoms is associated with increased risk of genital tract infection in the partners especially PID that may later lead to tubal obstruction and infertility. It has been reported that infertility in most low-income countries is from genital tract infection especially *Chlamydia trachomatis* leading to tubal infertility [15–17]. Women in Africa and Cameroon in particular seek care late, likely resulting in tubal disease from PID due to untreated Chlamydia infection [18]. Besides, a likely cause of infertility due to delayed presentation for treatment is age associated decline in ovarian reserve and egg quality due to aneuploidy [19, 20].

In this study, young age 15–25 years was protective of tubal infertility. This is consistent with the literature which states that women over 35 years generally have twice the risk of unexplained infertility, ovulatory dysfunction and tubal factor infertility [21] The study in Cameroon corroborates the fact that most infertile women were in the 30 to 40 years age bracket [9].

From the different studies, fertility may be multifactorial. However, our study population are women exposed early to sexually transmitted infections, coupled with the fact that they seek proper care relatively late [18].

### Reproductive health variables associated with tubal infertility

The protective association of condom use with infertility has consistently been reported in the literature [22–24]. Our study has shown that use of barrier contraception is protective of tubal infertility by preventing sexually transmitted infections and unwanted pregnancies. Voluntary termination of pregnancy is illegal in Cameroon. Therefore, most of these procedures are carried out in unorthodox conditions, sometimes by unqualified health or non-health professionals, resulting in increased rates of peritonitis, sepsis, and subsequent infertility [25–28].

The role of sexually transmitted infections in the genesis of fertility impairment is well documented and further corroborated in this study where *Chlamydia trachomatis* was 3.4-fold associated with tubal infertility [22–25]. In addition, an untreated STI is most often implicated in the development of PID and later fertility impairment [24, 25]. Furthermore, it has been reported previously that chronic active *Chlamydia* infection is often associated with tubal infertility, may persist despite therapy, and can be detected by endometrial biopsy culture [26].

Healthcare providers are in an excellent place not only to assess women at risk for STI but also to help them in decreasing their risks. Young women in particular should be targeted for screening and intervention on “fertility protection”. Of specific concern are the “silent” or atypical cases of PID that may present with vague symptoms but are not associated with pain; these infections are often only identified retrospectively during an infertility investigation [27, 28]. Unfortunately, there are no regular screening programmes for STI in Cameroon and in most cases only the antibody (serology) test for chlamydia is used.

There is also an increased prevalence of ectopic pregnancy likely due to undiagnosed or improperly treated STIs particularly *C. trachomatis*. The strength of the ectopic pregnancy variable speaks to the risks associated with undiagnosed and untreated sexually transmitted infections.

In this study the presence of uterine fibroids, especially when they are multiple, has been associated with tubal infertility. All our patients in Cameroon were of the black race. Therefore, they are prone to having uterine fibroids. It has been reported that uterine fibroids may be associated with tubal infertility (depending on the location of the fibroid) by local compression or occlusion of the tubal ostium or implantation failure. Submucosal fibroids had the strongest association with lower on-going pregnancy rates, primarily through decreased implantation. Cumulative pregnancy rates appeared slightly lower in patients with intramural fibroids. However, patients with intramural fibroids also experienced more miscarriages [29] . Besides, occasionally submucosal fibroids may cause tubal obstruction; more commonly unilateral [29, 30].

### Medical and surgical history associated with tubal infertility

In this study history of surgery for ectopic pregnancy and appendectomy has been associated with tubal infertility. However, infections that damage the tubes to cause ectopic gestation usually affect both tubes. In addition, surgery for ectopic pregnancy may lead to post-operative adhesions especially when done by open surgery or laparotomy [27, 31, 32]. That still, medical treatment of ectopic pregnancy with methotrexate has been reported in some studies in Cameroon and elsewhere [33–35]. This may lead to peritoneal adhesions that may impair fertility. Appendicitis may also lead to right tubal damage by contiguity thereby predisposing patients to pelvic peritonitis or ectopic pregnancy. However, several studies have reported that appendicitis is significantly associated to ectopic pregnancy and not to infertility [36–38]. However, a more recent study reported although previous appendectomy was associated with intra-abdominal adhesions, and these were in turn associated with tubal pathology, appendectomy was not directly associated with compromised tubal patency, but previous appendectomy may indirectly affect female fertility through mechanisms other than direct tubal obstruction [39].

Chronic pelvic pain was 7.3 times associated with tubal infertility. This is usually a corollary of organic damage to the genital tract and most often occurring after recurrent pelvic infections, chronic PID, pelvic surgery because of postoperative adhesions or endometriosis [40]. Endometriosis has been a silent cause of infertility in Cameroon because of peritoneal irritation, scarring and adhesion formation until the advent of laparoscopic surgery that brought clarity in its diagnosis.

In this study we found that diabetes mellitus has a 10.5-fold association with tubal infertility. Besides, one study reported that a history of infertility, particularly that related to ovulation disorders and tubal blockage, is significantly associated with a higher risk of type-2 diabetes mellitus [41].

Other factors like early coitarche (AOR = 2.93, *p* = 0.14), use of traditional herbs vaginally (AOR = 3.30, *p* = 0.23), multiple sexual partners (AOR = 2.40, *p* = 0.19) were not significant in the multivariate analysis. However, studies in Karachi-Pakistan have reported their roles in the genesis of tubal infertility [42].

### Prevention of tubal factor infertility

The practice of safe sex has been advocated as a means of reducing sexually transmitted infections (STI’s) and their sequelae that may lead to tubal infertility [43–45]. As already mentioned screening young girls for *Chlamydia trachomatis* and treating suspected cases of *C. trachomatis* infection is another important modality to avoid complications like pelvic inflammatory disease [27]. Furthermore, treatment of sexual partners of those infected will reduce reinfection among couples. However, caregivers who practice induced abortions should provide services to avoid post-abortal infections [46]. Finally, it is advisable to screen for *Chlamydia trachomatis* infection or administer prophylaxis against Chlamydia infection before invasive procedures like hysterosalpingography or hysteroscopy etcetera [47–49].

### Limitations and strengths of the study

In this study, we used the *Chlamydia* antibody test (serology) to test for chlamydia because it is less expensive although it is less specific in establishing an association with infertility. The presence of chlamydia antibodies does not confirm that chlamydia is the cause of the tubal pathology that led to infertility. However, the case-control design of this study reduced bias and the interpretative power of the risk factors of tubal infertility. Furthermore, the results of this study were based on information obtained from patient’s interviews, which could theoretically increase the risk of interviewer or responder bias as well as recall bias. Furthermore, we could not find out the compliance to treatment of these patients and their sexual practices during treatment for *Chlamydia trachomatis* infection that could lead to re-infection and chronicity. These may confound the outcome of treatment in favour of tubal damage and thereby accounting for the increase in tubal infertility reported in this study.

The use of in-depth patient interviews after a detailed explanation of study protocols limited recall bias. Additionally, infertile women and pregnant controls were motivated to report about earlier genital infections and other sexual problems that were requested from them because they believed that the researchers would educate them on their condition since these were sensitive issues. Besides, there is no significant difference between the two groups with regard to those under the age of 35, level of education, region of origin, and most professions aside from self-employed. This homogeneity of study population has made us to believe that the results of this study are valid and make an important contribution to risk factors of tubal infertility in Cameroon.

## Conclusion

The following factors were independently associated with tubal infertility: being a housewife, self-employed, history of *Chlamydia trachomatis* and *Mycoplasma* infection, and uterine fibroids. Furthermore, a history of pelvic surgery and other surgeries, diabetes mellitus, and chronic pelvic pain were also associated with tubal infertility. Young age, those in monogamous marriages, and users of barrier methods of contraception (condom) were less likely to have tubal infertility. Identification of these factors is a target of intervention to avoid tubal infertility.

## Data Availability

The data supporting the conclusions in this article is included in the article.

## Abbreviations

AOR: Adjusted odd ratio
C: Chlamydia
CI: Confidence interval
DRH: Douala Referral Hospital
HSG: Hysterosalpingography
PID: Pelvic Inflammatory Disease
SPSS: Statistical Package for the Social Sciences
STI: Sexually transmitted infection
